# Supplementary material for: Circulating Malondialdehyde Is a Potential Biomarker for Predicting All-Cause Mortality during Follow-Up by Reflecting Comprehensive Inflammation at Diagnosis in Patients with Antineutrophil Cytoplasmic Antibody-Associated Vasculitis
Source: Medicina (Kaunas). 2024 Jul 21;60(7):1182. doi: 10.3390/medicina60071182 (PMC11278744; doi:10.3390/medicina60071182)
Supplement: Supplementary file 1 [file medicina-60-01182-s001.zip › SUPPLEMENTARY TABLE S1(medicina-3082256)(1stREVISION).pdf]

Table S1. Comparison of variables between surviving and deceased patients with AAV

Variables	Surviving patients (N=72)	Deceased patients (N=6)	P-value
At diagnosis			
**Demographic data**			
Age (years)	62.5 (51.0−71.2))	74.5 (67.3−78.0)	0.028
Sex			0.221
Male sex (N, (%))	28 (38.9)	4 (66.7)	
Female sex (N, (%))	44 (61.1)	2 (33.3)	
Ex-smoker (N, (%))	3 (4.2)	0	1.000
Body mass index (kg/m^2)	22.4 (21.0−24.7)	22.7 (20.1−27.8)	0.771
**AAV subtypes (N, (%))**			0.185
MPA	33 (45.8)	5 (83.3)	
GPA	22 (30.6)	1 (16.7)	
EGPA	17 (23.6)	0 (0)	
**ANCA positivity (N, (%))**			
MPO-ANCA titre	0 (0−14.0)	79.5 (0−134.0)	0.067
PR3-ANCA titre	0 (0−0)	0 (0−0)	0.282
MPO-ANCA (or P-ANCA) positive	38 (52.8)	5 (83.3)	0.216
PR3-ANCA (or C-ANCA) positive	12 (16.7)	0 (0)	0.582
**AAV-specific indices**			
BVAS	5.0 (3.0−16.0)	18.0 (2.8−23.0)	0.222
FFS	0 (0−1.0)	1.5 (0−2.0)	0.137
**Comorbidities (N, (%))**			
Type 2 diabetes mellitus	14 (19.4)	3 (50.0)	0.114
Hypertension	23 (31.9)	2 (33.3)	1.000
**Acute-phase reactants**			
ESR (mm/h)	23.5 (8.3−68.0)	99.0 (22.5−120.0)	0.029
CRP (mg/L)	3.0 (0.7−13.7)	51.6 (2.5−81.4)	0.035
**cMDA (ng/mL)**	92.9 (0.6−191.1)	242.1 (77.7−359.8)	0.032
During follow-up			
**Medications**			
Glucocorticoids	71 (98.6)	6 (100)	1.000
Cyclophosphamide	46 (63.9)	5 (83.3)	0.658
Rituximab	13 (18.1)	3 (50.0)	0.097
Mycophenolate mofetil	19 (26.4)	1 (16.7)	1.000
Azathioprine	46 (63.9)	2 (33.3)	0.197
Tacrolimus	6 (8.3)	1 (16.7)	0.442
Methotrexate	3 (4.2)	0 (0)	1.000

Values are expressed as a median (interquartile range [IQR]) or N (%).
AAV: ANCA-associated vasculitis; ANCA: antineutrophil cytoplasmic antibody; MPA: microscopic polyangiitis; GPA: granulomatosis with polyangiitis; MPO: myeloperoxidase; P: perinuclear; PR3: proteinase 3; C: cytoplasmic; BVAS: the Birmingham vasculitis activity score; FFS: the five-factor score; ESR: erythrocyte sedimentation rate; CRP: C-reactive protein; cMDA: circulating malondialdehyde.
