# Supplementary material for: Circulating Malondialdehyde Is a Potential Biomarker for Predicting All-Cause Mortality during Follow-Up by Reflecting Comprehensive Inflammation at Diagnosis in Patients with Antineutrophil Cytoplasmic Antibody-Associated Vasculitis
Source: Medicina (Kaunas). 2024 Jul 21;60(7):1182. doi: 10.3390/medicina60071182 (PMC11278744; doi:10.3390/medicina60071182)
Supplement: Supplementary file 1 [file medicina-60-01182-s001.zip › SUPPLEMENTARY TABLE S2(medicina-3082256)(1stREVISION).pdf]

Table S2. Comparison of variables between patients with cMDA ≥221.7 ng/mL and those with cMDA <221.7 ng/mL at diagnosis

Variables	patients with cMDA <221.7 ng/mL at diagnosis (N=64)	patients with cMDA ≥221.7 ng/mL at diagnosis (N=14)	P-value
At diagnosis			
**Demographic data**			
Age (years)	63.0 (51.3−72.7)	65.5 (51.8−76.3)	0.470
Sex			0.451
Male sex (N, (%))	39 (60.9)	7 (50.0)	
Female sex (N, (%))	25 (39.1)	7 (50.0)	
Ex-smoker (N, (%))	3 (4.7)	0 (0)	1.000
Body mass index (kg/m^2)	22.4 (20.9−24.7)	22.4 (20.2−24.9)	0.943
**AAV subtypes (N, (%))**			0.731
MPA	30 (46.9)	8 (57.1)	
GPA	20 (31.3)	3 (21.4)	
EGPA	14 (21.9)	3 (21.4)	
**ANCA positivity (N, (%))**			
MPO-ANCA titre	0 (0−15.0)	0 (0−127.3)	0.895
PR3-ANCA titre	0 (0−0)	0 (0−0)	0.983
MPO-ANCA (or P-ANCA) positive	34 (53.1)	9 (64.3)	0.447
PR3-ANCA (or C-ANCA) positive	10 (15.6)	2 (14.3)	1.000
**AAV-specific indices**			
BVAS	5.0 (3.0−17.0)	4.0 (1.8−18.5)	0.544
FFS	0 (0−1.0)	1.0 (0−2.0)	0.281
**Comorbidities (N, (%))**			
Type 2 diabetes mellitus	14 (21.9)	3 (21.4)	1.000
Hypertension	21 (32.8)	4 (28.6)	1.000
**Acute-phase reactants**			
ESR (mm/h)	22.5 (7.3−75.5)	44.0 (16.8−105.0)	0.091
CRP (mg/L)	3.5 (0.7−13.6)	3.3 (1.2−59.2)	0.310
**cMDA (ng/mL)**	50.3 (5.2−146.9)	262.2 (248.1−353.8)	<0.001
During follow-up			
**Mortality**			
All-cause mortality	2 (3.1)	4 (28.6)	0.008
Follow-up duration based on all-cause mortality	27.9 (12.6−46.5)	26.3 (5.0−38.8)	0.274
**Medications**			
Glucocorticoids	63 (98.4)	14 (100	1.000
Cyclophosphamide	40 (62.5)	11 (78.6)	0.357
Rituximab	12 (18.8)	4 (28.6)	0.469
Mycophenolate mofetil	15 (23.4)	5 (35.7)	0.341
Azathioprine	40 (62.5)	8 (57.1)	0.709
Tacrolimus	5 (7.8)	2 (14.3)	0.603
Methotrexate	2 (3.1)	1 (7.1)	0.452

Values are expressed as a median (25~75 percentile) or N (%).

cMDA: circulating malondialdehyde; AAV: ANCA-associated vasculitis; ANCA: antineutrophil cytoplasmic antibody; MPA: microscopic polyangiitis; GPA: granulomatosis with polyangiitis; MPO: myeloperoxidase; P: perinuclear; PR3: proteinase 3; C: cytoplasmic; BVAS: the Birmingham vasculitis activity score; FFS: the five-factor score; ESR: erythrocyte sedimentation rate; CRP: C-reactive protein.
