# Supplementary figures and images for: Circulating Malondialdehyde Is a Potential Biomarker for Predicting All-Cause Mortality during Follow-Up by Reflecting Comprehensive Inflammation at Diagnosis in Patients with Antineutrophil Cytoplasmic Antibody-Associated Vasculitis
Source: Medicina (Kaunas). 2024 Jul 21;60(7):1182. doi: 10.3390/medicina60071182 (PMC11278744; doi:10.3390/medicina60071182)

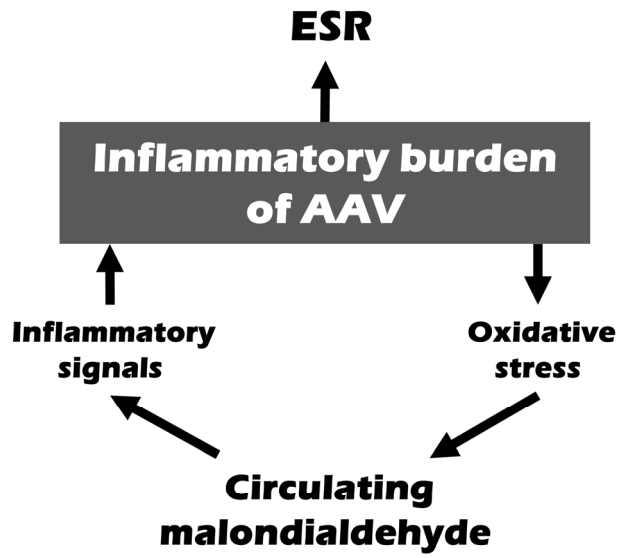

**Figure S1:** Inferences on the hypothesis of the clinical utility of cMDA in patients with AAV.

Supplement: Supplementary file 1 [file medicina-60-01182-s001.zip › Figure S1.pdf]
